# (*E*)-4-Meth­oxy-*N*′-(2,4,5-tri­meth­oxy­benzyl­idene)benzohydrazide hemihydrate

**DOI:** 10.1107/S1600536814000531

**Published:** 2014-01-15

**Authors:** Suchada Chantrapromma, Nawong Boonnak, Jirapa Horkaew, Ching Kheng Quah, Hoong-Kun Fun

**Affiliations:** a Department of Chemistry, Faculty of Science, Prince of Songkla University, Hat-Yai, Songkhla 90112, Thailand; b Faculty of Traditional Thai Medicine, Prince of Songkla University, Hat-Yai, Songkhla 90112, Thailand; c X-ray Crystallography Unit, School of Physics, Universiti Sains Malaysia, 11800 USM, Penang, Malaysia

## Abstract

The title compound crystallizes as a hemihydrate, C_18_H_20_N_2_O_5_·0.5H_2_O. The mol­ecule exists in an *E* conformation with respect to the C=N imine bond. The 4-meth­oxy­phenyl unit is disordered over two sets of sites with a refined occupancy ratio of 0.54 (2):0.46 (2). The dihedral angles between the benzene rings are 29.20 (9) and 26.59 (9)°, respectively, for the major and minor components of the 4-meth­oxy-substituted ring. All meth­oxy substituents lie close to the plane of the attached benzene rings [the C_meth­yl_—O—C—C torsion angles range from −4.0 (12) to 3.9 (2)°]. In the crystal, the components are linked into chains propagating along [001] *via* N—H⋯O and O—H⋯O hydrogen bonds and weak C—H⋯O inter­actions.

## Related literature   

For standard bond-length data, see: Allen *et al.* (1987 ▶). For related structures, see: Fun *et al.* (2012 ▶); Horkaew *et al.* (2011 ▶). For applications of benzohydrazide derivatives, see: Molyneux (2004 ▶); Raj *et al.* (2007 ▶); Sathyadevi *et al.* (2012 ▶); Wang *et al.* (2012 ▶). For the stability of the temperature controller used in the data collection, see: Cosier & Glazer (1986 ▶).
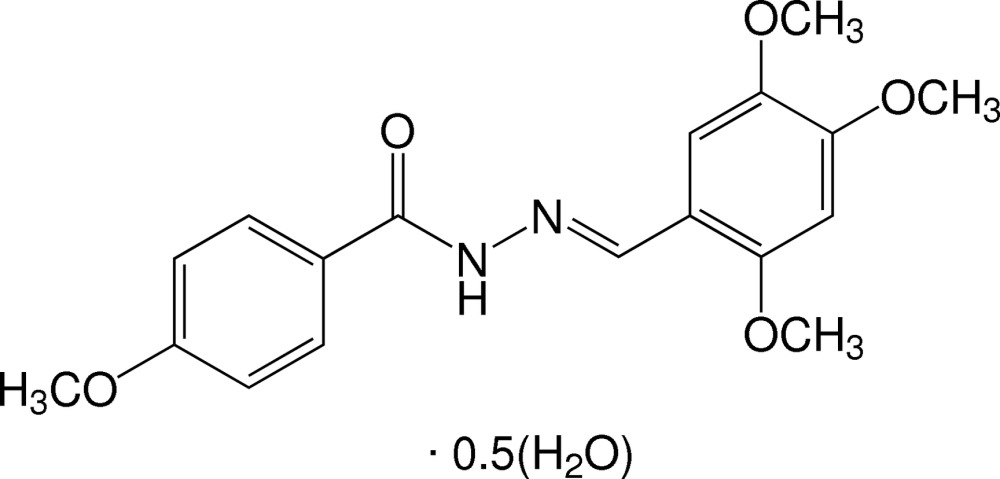


## Experimental   

### 

#### Crystal data  

C_18_H_20_N_2_O_5_·0.5H_2_O
*M* *_r_* = 353.37
Monoclinic, 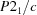

*a* = 13.4405 (3) Å
*b* = 16.9172 (3) Å
*c* = 7.6841 (2) Å
β = 96.084 (1)°
*V* = 1737.34 (7) Å^3^
*Z* = 4
Mo *K*α radiation
μ = 0.10 mm^−1^
*T* = 100 K
0.28 × 0.18 × 0.08 mm

#### Data collection  

Bruker SMART APEXII CCD area-detector diffractometer
Absorption correction: multi-scan (*SADABS*; Bruker, 2009 ▶) *T*_min_ = 0.972, *T*_max_ = 0.992
13808 measured reflections
4606 independent reflections
2993 reflections with *I* > 2σ(*I*)
*R*_int_ = 0.044

#### Refinement  

*R*[*F* ^2^ > 2σ(*F* ^2^)] = 0.059
*wR*(*F* ^2^) = 0.138
*S* = 1.04
4606 reflections
293 parameters
264 restraints
H atoms treated by a mixture of independent and constrained refinement
Δρ_max_ = 0.34 e Å^−3^
Δρ_min_ = −0.31 e Å^−3^

### 

Data collection: *APEX2* (Bruker, 2009 ▶); cell refinement: *SAINT* (Bruker, 2009 ▶); data reduction: *SAINT*; program(s) used to solve structure: *SHELXTL* (Sheldrick, 2008 ▶); program(s) used to refine structure: *SHELXTL*; molecular graphics: *SHELXTL*; software used to prepare material for publication: *SHELXTL*, *PLATON* (Spek, 2009 ▶) and *publCIF* (Westrip, 2010 ▶).

## Supplementary Material

Crystal structure: contains datablock(s) global, I. DOI: 10.1107/S1600536814000531/lh5678sup1.cif

Structure factors: contains datablock(s) I. DOI: 10.1107/S1600536814000531/lh5678Isup2.hkl

Supporting information file. DOI: 10.1107/S1600536814000531/lh5678Isup3.cml

CCDC reference:

Additional supporting information:  crystallographic information; 3D view; checkCIF report

## Figures and Tables

**Table 1:** Hydrogen-bond geometry (Å, °)

*D*—H⋯*A*	*D*—H	H⋯*A*	*D*⋯*A*	*D*—H⋯*A*
N1—H1*N*1⋯O1^i^	0.89 (2)	1.94 (2)	2.8086 (19)	166 (2)
O1*W*—H2*W*1⋯O5^ii^	0.85	2.36	3.068 (4)	141
O1*W*—H1*W*1⋯O4^ii^	0.85	2.33	3.036 (5)	141
C6*A*—H6*BA*⋯O1^i^	0.93	2.55	3.294 (17)	138
C8—H8*A*⋯O1^i^	0.93	2.49	3.2786 (19)	143

Symmetry codes: (i) 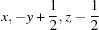; (ii) 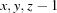.

**Fig. 1.**
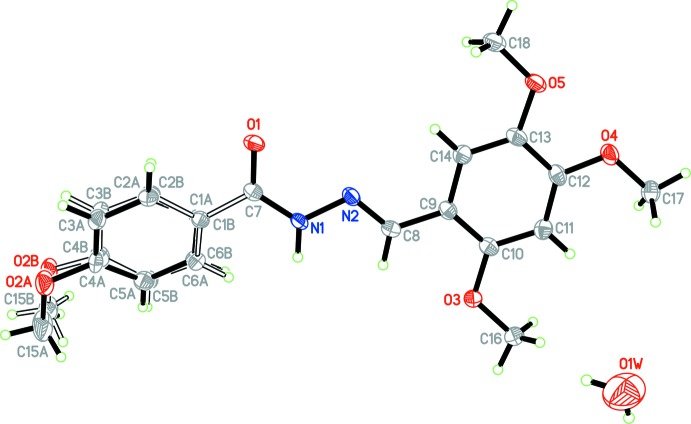
The molecular structure of (I) showing 60% probability displacement ellipsoids. Open bonds show the minor component.

**Fig. 2.**
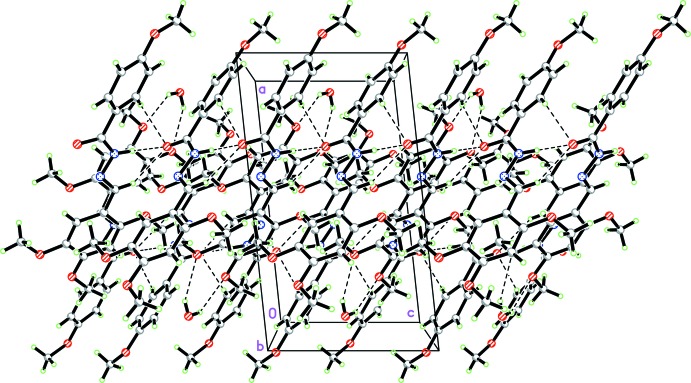
The crystal packing viewed along the b axis showing hydrogen bonds drawn as dashed lines. Only the major component of disorder is shown.

## supplementary crystallographic information

### 1. Comment

Benzohydrazide derivatives and their complexes have been found to exhibit various biological properties, such as analgesic, antifungal and antibacterial (Raj *et al.*, 2007 and Wang *et al.*, 2012 ), including antioxidant and biocidal activities (Sathyadevi *et al.*, 2012). In contiuation of our on-going research on the bioactivity of benzohydrazides, the title compound (I) was synthesized and evaluated for antioxidant activity by DPPH free radical scavenging method (Molyneux, 2004), but was found to be inactive. It was also screened for antibacterial activity against *B. subtilis*, *S. aureus*, *P. aeruginosa*, *S. typhi* and *S. sonnei* and also found to be inactive. Herein we report the synthesis and crystal structure of (I).

The asymmetric unit of (I) (Fig. 1) consists of a C_18_H_20_N_2_O_5_ molecule and half an H_2_O molecule. The 4-methoxyphenyl unit is disordered over two positions with a refined site-occupancy ratio of 0.538 (2):0.462 (2). The benzohydrazide exists in an *E* configuration with respect to the C8═N2 imine bond [1.285 (2)Å] and the N1—N2—C8—C9 torsion angle is 178.38 (15)°. The molecule is twisted with a dihedral angle between the two substituted benzene rings being 29.20 (9) and 26.59 (9)° for the major *A* and minor *B* components, respectively. Five atoms (O1, C7, N1, N2 and C8) of the middle bridge fragament lie on the same plane with the *r.m.s.* deviation of 0.0187 (2) Å. The mean plane through this middle fragment makes the dihedral angles of 22.67 (9) and 19.51 (9)° with the C1–C6 benzene ring for the major *A* and minor *B* components, respectively, and 6.84 (10)° with the C9–C14 benzene ring. The methoxy substituent of 4-methoxyphenyl lies close to the plane of the attached benzene ring with the torsion angle C15–O2–C4–C5 = 0.1 (11)° and the *r.m.s.* deviation of 0.0236 (2) Å for the eight non-H atoms of 4-methoxyphenyl moiety for the major *A* component [the corresponding values are -4.0 (12)° and 0.0210 (2) Å for the minor *B* component]. The three methoxy substituents of 2,4,5-trimethoxyphenyl unit are essentially co-planar with the bound benzene rings with the *r.m.s.* deviation of 0.0113 (2) Å for the twelve non-H atoms, and the torsion angles C16–O3–C10–C11 = 3.9 (3)°, C17–O4–C12–C13 = -179.46 (15)° and C18–O5–C13–C14 = -0.6 (3)°. These torsion angles indicated that the methoxy group at the *para*-position or at atom C12 points towards an opposite direction with the other two at the *ortho*-position or at atom C10 and the *meta*-position or at atom C13 (Fig. 1). Bond distances are of normal values (Allen *et al.*, 1987) and are comparable with the closely related structures (Fun *et al.*, 2012 and Horkaew *et al.*, 2011).

In the crystal packing (Fig. 2), the benzohydrazide and water molecules are linked by O—H···O, N—H···O hydrogen bonds and weak C—H···O interactions (Table 1) into chains along [0 0 1]. The crystal is consolidated by these interactions.

### 2. Experimental

The title compound (I) was prepared by dissolving 4-methoxybenzohydrazide (2 mmol, 0.30 g) in ethanol (10 ml). The solution of 2,4,5-trimethoxybenzaldehyde (2 mmol, 0.40 g) in ethanol (10 ml) was then added slowly to commence the reaction. The reaction mixture was refluxed for around 3 hr. The solution was then cooled to room temperature yielding a white solid, which was collected by filtration, washed with ethanol and dried in air. Colorless plate-shaped single crystals of the title compound suitable for *X*-ray structure determination were recrystallized from ethanol by slow evaporation of the solvent at room temperature after several days.

### 3. Refinement

The amide H atom was located a the difference map was refined freely. The remaining H atoms were positioned geometrically and allowed to ride on their parent atoms, with d(O—H) = 0.85 Å, d(C—H) = 0.93 Å for aromatic and C—H and 0.96 Å for CH_3_ atoms. The *U*_iso_ values were constrained to be 1.5*U*_eq_ of the carrier atom for methyl H atoms and 1.2*U*_eq_ for the remaining H atoms. A rotating group model was used for the methyl groups. The 4-methoxyphenyl unit is disordered over two sites with refined site occupancies ratio 0.538 (2):0.462 (2). Similarity and simulation restraints were applied. The thermal ellipsoids of each pair of atoms i.e. "C1A C1B", "C2A C2B", "C5A C5B" and "C6A C6B" were restrained to be equal.

### Crystal data

**Table:**

C_18_H_20_N_2_O_5_·0.5H_2_O	*F*(000) = 748
*M**_r_* = 353.37	*D*_x_ = 1.351 Mg m^−^^3^
Monoclinic, *P*2_1_/*c*	Mo *K*α radiation, λ = 0.71073 Å
Hall symbol: -P 2ybc	Cell parameters from 4606 reflections
*a* = 13.4405 (3) Å	θ = 1.9–29.0°
*b* = 16.9172 (3) Å	µ = 0.10 mm^−^^1^
*c* = 7.6841 (2) Å	*T* = 100 K
β = 96.084 (1)°	Plate, colorless
*V* = 1737.34 (7) Å^3^	0.28 × 0.18 × 0.08 mm
*Z* = 4	

### Data collection

**Table:**

Bruker SMART APEXII CCD area-detector diffractometer	4606 independent reflections
Radiation source: fine-focus sealed tube	2993 reflections with *I* > 2σ(*I*)
Graphite monochromator	*R*_int_ = 0.044
Detector resolution: 8.33 pixels mm^-1^	θ_max_ = 29.0°, θ_min_ = 1.9°
ω scans	*h* = −17→18
Absorption correction: multi-scan (*SADABS*; Bruker, 2009)	*k* = −20→23
*T*_min_ = 0.972, *T*_max_ = 0.992	*l* = −10→10
13808 measured reflections	

### Refinement

**Table:**

Refinement on *F*^2^	Primary atom site location: structure-invariant direct methods
Least-squares matrix: full	Secondary atom site location: difference Fourier map
*R*[*F*^2^ > 2σ(*F*^2^)] = 0.059	Hydrogen site location: inferred from neighbouring sites
*wR*(*F*^2^) = 0.138	H atoms treated by a mixture of independent and constrained refinement
*S* = 1.04	*w* = 1/[σ^2^(*F*_o_^2^) + (0.0568*P*)^2^ + 0.440*P*] where *P* = (*F*_o_^2^ + 2*F*_c_^2^)/3
4606 reflections	(Δ/σ)_max_ = 0.001
293 parameters	Δρ_max_ = 0.34 e Å^−^^3^
264 restraints	Δρ_min_ = −0.31 e Å^−^^3^

### Special details

**Table:**

Experimental. The data was collected with the Oxford Cyrosystem Cobra low-temperature attachment.
Geometry. All esds (except the esd in the dihedral angle between two l.s. planes) are estimated using the full covariance matrix. The cell esds are taken into account individually in the estimation of esds in distances, angles and torsion angles; correlations between esds in cell parameters are only used when they are defined by crystal symmetry. An approximate (isotropic) treatment of cell esds is used for estimating esds involving l.s. planes.
Refinement. Refinement of F^2^ against ALL reflections. The weighted R-factor wR and goodness of fit S are based on F^2^, conventional R-factors R are based on F, with F set to zero for negative F^2^. The threshold expression of F^2^ > 2sigma(F^2^) is used only for calculating R-factors(gt) etc. and is not relevant to the choice of reflections for refinement. R-factors based on F^2^ are statistically about twice as large as those based on F, and R- factors based on ALL data will be even larger.

### Fractional atomic coordinates and isotropic or equivalent isotropic displacement parameters (Å^2^)

**Table:**

	*x*	*y*	*z*	*U*_iso_*/*U*_eq_	Occ. (<1)
O1	0.29968 (9)	0.15688 (7)	1.03678 (15)	0.0175 (3)	
O3	0.55282 (9)	0.46403 (8)	0.75008 (15)	0.0213 (3)	
O4	0.78081 (9)	0.49186 (8)	1.28062 (16)	0.0217 (3)	
O5	0.69285 (10)	0.37759 (8)	1.42709 (16)	0.0244 (3)	
N1	0.32902 (11)	0.25582 (9)	0.8489 (2)	0.0163 (3)	
H1N1	0.3183 (16)	0.2756 (13)	0.741 (3)	0.033 (6)*	
N2	0.41000 (11)	0.28402 (9)	0.95977 (18)	0.0166 (3)	
O2A	−0.0765 (6)	0.0960 (5)	0.4836 (12)	0.0297 (14)	0.54 (2)
C1A	0.1810 (10)	0.1723 (12)	0.787 (2)	0.0149 (6)	0.54 (2)
C2A	0.1424 (9)	0.0957 (10)	0.8016 (18)	0.0163 (10)	0.54 (2)
H2BA	0.1757	0.0602	0.8798	0.020*	0.54 (2)
C3A	0.0560 (8)	0.0726 (7)	0.7023 (13)	0.0183 (15)	0.54 (2)
H3BA	0.0312	0.0218	0.7142	0.022*	0.54 (2)
C4A	0.0057 (6)	0.1252 (6)	0.5839 (14)	0.0202 (15)	0.54 (2)
C5A	0.0422 (10)	0.2013 (7)	0.569 (2)	0.0203 (7)	0.54 (2)
H5BA	0.0086	0.2369	0.4910	0.024*	0.54 (2)
C6A	0.1292 (12)	0.2241 (10)	0.670 (3)	0.0159 (8)	0.54 (2)
H6BA	0.1532	0.2753	0.6597	0.019*	0.54 (2)
C15A	−0.1282 (7)	0.1466 (6)	0.3551 (17)	0.043 (2)	0.54 (2)
H15D	−0.1855	0.1194	0.2988	0.065*	0.54 (2)
H15E	−0.0842	0.1607	0.2694	0.065*	0.54 (2)
H15F	−0.1495	0.1935	0.4107	0.065*	0.54 (2)
O2B	−0.0920 (6)	0.1119 (6)	0.5300 (14)	0.0279 (16)	0.46 (2)
C1B	0.1849 (12)	0.1741 (14)	0.783 (3)	0.0149 (6)	0.46 (2)
C2B	0.1382 (10)	0.1019 (12)	0.816 (2)	0.0163 (10)	0.46 (2)
H2AA	0.1695	0.0664	0.8959	0.020*	0.46 (2)
C3B	0.0463 (9)	0.0839 (8)	0.7286 (16)	0.023 (2)	0.46 (2)
H3AA	0.0154	0.0363	0.7508	0.028*	0.46 (2)
C4B	−0.0008 (8)	0.1368 (8)	0.6073 (17)	0.0228 (18)	0.46 (2)
C5B	0.0434 (12)	0.2084 (8)	0.575 (2)	0.0203 (7)	0.46 (2)
H5AA	0.0117	0.2440	0.4952	0.024*	0.46 (2)
C6B	0.1360 (14)	0.2261 (12)	0.664 (3)	0.0159 (8)	0.46 (2)
H6AA	0.1660	0.2742	0.6431	0.019*	0.46 (2)
C15B	−0.1472 (7)	0.1666 (6)	0.4161 (15)	0.034 (2)	0.46 (2)
H15A	−0.2100	0.1434	0.3716	0.051*	0.46 (2)
H15B	−0.1096	0.1793	0.3204	0.051*	0.46 (2)
H15C	−0.1592	0.2139	0.4796	0.051*	0.46 (2)
C7	0.27597 (13)	0.19396 (10)	0.8996 (2)	0.0150 (4)	
C8	0.45543 (12)	0.34220 (10)	0.8953 (2)	0.0157 (4)	
H8A	0.4335	0.3599	0.7832	0.019*	
C9	0.54027 (13)	0.38105 (10)	0.9932 (2)	0.0158 (4)	
C10	0.58893 (13)	0.44309 (10)	0.9176 (2)	0.0164 (4)	
C11	0.67034 (13)	0.48128 (11)	1.0102 (2)	0.0180 (4)	
H11A	0.7027	0.5222	0.9581	0.022*	
C12	0.70267 (13)	0.45794 (11)	1.1801 (2)	0.0176 (4)	
C13	0.65430 (13)	0.39560 (11)	1.2589 (2)	0.0184 (4)	
C14	0.57447 (13)	0.35826 (10)	1.1662 (2)	0.0175 (4)	
H14A	0.5424	0.3172	1.2185	0.021*	
C16	0.60554 (14)	0.52397 (12)	0.6663 (2)	0.0235 (4)	
H16A	0.5719	0.5343	0.5521	0.035*	
H16B	0.6725	0.5064	0.6556	0.035*	
H16C	0.6076	0.5715	0.7350	0.035*	
C17	0.83064 (14)	0.55583 (11)	1.2041 (2)	0.0209 (4)	
H17A	0.8844	0.5746	1.2859	0.031*	
H17B	0.7839	0.5980	1.1752	0.031*	
H17C	0.8570	0.5378	1.0998	0.031*	
C18	0.64432 (16)	0.31540 (13)	1.5103 (3)	0.0299 (5)	
H18A	0.6775	0.3071	1.6257	0.045*	
H18B	0.6470	0.2678	1.4430	0.045*	
H18C	0.5757	0.3295	1.5181	0.045*	
O1W	0.9194 (3)	0.3907 (3)	0.5262 (5)	0.0688 (12)	0.50
H2W1	0.8632	0.3689	0.5354	0.103*	0.50
H1W1	0.9088	0.4310	0.4608	0.103*	0.50

### Atomic displacement parameters (Å^2^)

**Table:**

	*U*^11^	*U*^22^	*U*^33^	*U*^12^	*U*^13^	*U*^23^
O1	0.0241 (7)	0.0158 (6)	0.0120 (6)	0.0003 (6)	−0.0013 (5)	0.0002 (5)
O3	0.0216 (7)	0.0246 (7)	0.0166 (7)	−0.0069 (6)	−0.0028 (5)	0.0070 (6)
O4	0.0245 (7)	0.0180 (7)	0.0211 (7)	−0.0073 (6)	−0.0048 (5)	0.0000 (6)
O5	0.0314 (7)	0.0232 (7)	0.0163 (7)	−0.0086 (6)	−0.0079 (5)	0.0035 (6)
N1	0.0190 (8)	0.0170 (8)	0.0118 (7)	−0.0027 (7)	−0.0037 (6)	0.0008 (6)
N2	0.0177 (7)	0.0174 (8)	0.0139 (7)	−0.0015 (7)	−0.0017 (6)	−0.0021 (6)
O2A	0.023 (2)	0.030 (3)	0.033 (3)	−0.0094 (18)	−0.0111 (19)	0.008 (2)
C1A	0.0169 (11)	0.0155 (12)	0.0127 (9)	−0.0008 (9)	0.0028 (9)	−0.0026 (8)
C2A	0.0180 (11)	0.016 (2)	0.0148 (18)	0.0016 (11)	0.0013 (12)	0.0020 (15)
C3A	0.020 (2)	0.013 (3)	0.021 (3)	−0.002 (2)	0.003 (2)	0.000 (2)
C4A	0.017 (2)	0.020 (3)	0.023 (3)	−0.009 (2)	−0.0001 (19)	0.001 (2)
C5A	0.0193 (9)	0.0210 (18)	0.0199 (12)	0.0010 (11)	−0.0009 (8)	0.0044 (14)
C6A	0.0162 (17)	0.0156 (11)	0.0162 (13)	−0.0025 (10)	0.0032 (13)	0.0015 (9)
C15A	0.032 (3)	0.039 (4)	0.052 (5)	−0.013 (3)	−0.025 (3)	0.018 (4)
O2B	0.020 (2)	0.029 (3)	0.032 (3)	−0.010 (2)	−0.010 (2)	0.010 (2)
C1B	0.0169 (11)	0.0155 (12)	0.0127 (9)	−0.0008 (9)	0.0028 (9)	−0.0026 (8)
C2B	0.0180 (11)	0.016 (2)	0.0148 (18)	0.0016 (11)	0.0013 (12)	0.0020 (15)
C3B	0.022 (3)	0.023 (4)	0.026 (3)	−0.008 (3)	0.002 (3)	0.001 (3)
C4B	0.018 (2)	0.026 (3)	0.023 (3)	−0.002 (2)	−0.004 (2)	0.004 (3)
C5B	0.0193 (9)	0.0210 (18)	0.0199 (12)	0.0010 (11)	−0.0009 (8)	0.0044 (14)
C6B	0.0162 (17)	0.0156 (11)	0.0162 (13)	−0.0025 (10)	0.0032 (13)	0.0015 (9)
C15B	0.024 (3)	0.039 (4)	0.037 (4)	−0.012 (3)	−0.012 (3)	0.012 (3)
C7	0.0175 (8)	0.0145 (9)	0.0132 (8)	0.0023 (7)	0.0022 (6)	−0.0040 (7)
C8	0.0170 (8)	0.0167 (9)	0.0133 (8)	0.0021 (8)	0.0004 (6)	0.0002 (7)
C9	0.0166 (8)	0.0148 (9)	0.0155 (9)	0.0013 (7)	−0.0001 (6)	−0.0022 (7)
C10	0.0180 (9)	0.0147 (9)	0.0159 (9)	0.0033 (7)	−0.0008 (7)	−0.0001 (7)
C11	0.0181 (9)	0.0146 (9)	0.0212 (9)	−0.0003 (8)	0.0019 (7)	0.0006 (8)
C12	0.0176 (9)	0.0145 (9)	0.0199 (9)	−0.0005 (7)	−0.0018 (7)	−0.0038 (7)
C13	0.0232 (9)	0.0176 (9)	0.0136 (9)	0.0007 (8)	−0.0019 (7)	−0.0024 (8)
C14	0.0202 (9)	0.0135 (9)	0.0186 (9)	−0.0005 (8)	0.0013 (7)	0.0002 (7)
C16	0.0239 (10)	0.0267 (11)	0.0194 (10)	−0.0063 (9)	0.0004 (8)	0.0054 (8)
C17	0.0220 (10)	0.0163 (10)	0.0244 (10)	−0.0040 (8)	0.0013 (8)	−0.0029 (8)
C18	0.0416 (12)	0.0283 (11)	0.0175 (10)	−0.0129 (10)	−0.0073 (8)	0.0045 (9)
O1W	0.059 (2)	0.083 (3)	0.064 (3)	0.011 (2)	0.006 (2)	0.031 (2)

### Geometric parameters (Å, º)

**Table:**

O1—C7	1.239 (2)	C2B—C3B	1.374 (6)
O3—C10	1.373 (2)	C2B—H2AA	0.9300
O3—C16	1.429 (2)	C3B—C4B	1.395 (6)
O4—C12	1.363 (2)	C3B—H3AA	0.9300
O4—C17	1.431 (2)	C4B—C5B	1.383 (6)
O5—C13	1.375 (2)	C5B—C6B	1.388 (6)
O5—C18	1.425 (2)	C5B—H5AA	0.9300
N1—C7	1.347 (2)	C6B—H6AA	0.9300
N1—N2	1.393 (2)	C15B—H15A	0.9600
N1—H1N1	0.89 (2)	C15B—H15B	0.9600
N2—C8	1.285 (2)	C15B—H15C	0.9600
O2A—C4A	1.370 (5)	C8—C9	1.455 (2)
O2A—C15A	1.430 (5)	C8—H8A	0.9300
C1A—C6A	1.387 (5)	C9—C10	1.395 (3)
C1A—C2A	1.404 (5)	C9—C14	1.413 (2)
C1A—C7	1.511 (7)	C10—C11	1.398 (2)
C2A—C3A	1.377 (5)	C11—C12	1.389 (2)
C2A—H2BA	0.9300	C11—H11A	0.9300
C3A—C4A	1.395 (5)	C12—C13	1.409 (3)
C3A—H3BA	0.9300	C13—C14	1.377 (2)
C4A—C5A	1.388 (5)	C14—H14A	0.9300
C5A—C6A	1.389 (5)	C16—H16A	0.9600
C5A—H5BA	0.9300	C16—H16B	0.9600
C6A—H6BA	0.9300	C16—H16C	0.9600
C15A—H15D	0.9600	C17—H17A	0.9600
C15A—H15E	0.9600	C17—H17B	0.9600
C15A—H15F	0.9600	C17—H17C	0.9600
O2B—C4B	1.371 (5)	C18—H18A	0.9600
O2B—C15B	1.427 (6)	C18—H18B	0.9600
C1B—C6B	1.384 (6)	C18—H18C	0.9600
C1B—C2B	1.408 (6)	O1W—H2W1	0.8500
C1B—C7	1.475 (9)	O1W—H1W1	0.8500
			
C10—O3—C16	117.55 (14)	O2B—C15B—H15C	109.5
C12—O4—C17	116.85 (14)	H15A—C15B—H15C	109.5
C13—O5—C18	116.14 (14)	H15B—C15B—H15C	109.5
C7—N1—N2	119.49 (15)	O1—C7—N1	122.91 (16)
C7—N1—H1N1	121.5 (14)	O1—C7—C1B	121.5 (9)
N2—N1—H1N1	118.6 (14)	N1—C7—C1B	115.6 (9)
C8—N2—N1	113.50 (15)	O1—C7—C1A	119.5 (8)
C4A—O2A—C15A	118.5 (5)	N1—C7—C1A	117.6 (8)
C6A—C1A—C2A	118.2 (5)	N2—C8—C9	121.65 (16)
C6A—C1A—C7	123.6 (11)	N2—C8—H8A	119.2
C2A—C1A—C7	118.2 (11)	C9—C8—H8A	119.2
C3A—C2A—C1A	120.9 (6)	C10—C9—C14	118.47 (16)
C3A—C2A—H2BA	119.6	C10—C9—C8	120.03 (16)
C1A—C2A—H2BA	119.6	C14—C9—C8	121.49 (16)
C2A—C3A—C4A	120.2 (5)	O3—C10—C9	116.72 (15)
C2A—C3A—H3BA	119.9	O3—C10—C11	122.50 (16)
C4A—C3A—H3BA	119.9	C9—C10—C11	120.77 (16)
O2A—C4A—C5A	123.9 (6)	C12—C11—C10	119.79 (17)
O2A—C4A—C3A	116.4 (5)	C12—C11—H11A	120.1
C5A—C4A—C3A	119.7 (5)	C10—C11—H11A	120.1
C4A—C5A—C6A	119.6 (6)	O4—C12—C11	123.95 (16)
C4A—C5A—H5BA	120.2	O4—C12—C13	115.79 (16)
C6A—C5A—H5BA	120.2	C11—C12—C13	120.25 (16)
C1A—C6A—C5A	121.4 (6)	O5—C13—C14	125.45 (16)
C1A—C6A—H6BA	119.3	O5—C13—C12	115.12 (15)
C5A—C6A—H6BA	119.3	C14—C13—C12	119.43 (16)
C4B—O2B—C15B	116.7 (6)	C13—C14—C9	121.28 (17)
C6B—C1B—C2B	118.5 (6)	C13—C14—H14A	119.4
C6B—C1B—C7	124.2 (13)	C9—C14—H14A	119.4
C2B—C1B—C7	116.6 (13)	O3—C16—H16A	109.5
C3B—C2B—C1B	120.1 (7)	O3—C16—H16B	109.5
C3B—C2B—H2AA	119.9	H16A—C16—H16B	109.5
C1B—C2B—H2AA	119.9	O3—C16—H16C	109.5
C2B—C3B—C4B	120.2 (6)	H16A—C16—H16C	109.5
C2B—C3B—H3AA	119.9	H16B—C16—H16C	109.5
C4B—C3B—H3AA	119.9	O4—C17—H17A	109.5
O2B—C4B—C5B	124.9 (7)	O4—C17—H17B	109.5
O2B—C4B—C3B	114.5 (7)	H17A—C17—H17B	109.5
C5B—C4B—C3B	120.6 (6)	O4—C17—H17C	109.5
C4B—C5B—C6B	118.7 (7)	H17A—C17—H17C	109.5
C4B—C5B—H5AA	120.7	H17B—C17—H17C	109.5
C6B—C5B—H5AA	120.7	O5—C18—H18A	109.5
C1B—C6B—C5B	121.9 (7)	O5—C18—H18B	109.5
C1B—C6B—H6AA	119.1	H18A—C18—H18B	109.5
C5B—C6B—H6AA	119.1	O5—C18—H18C	109.5
O2B—C15B—H15A	109.5	H18A—C18—H18C	109.5
O2B—C15B—H15B	109.5	H18B—C18—H18C	109.5
H15A—C15B—H15B	109.5	H2W1—O1W—H1W1	107.7
			
C7—N1—N2—C8	178.86 (15)	C2B—C1B—C7—C1A	−37 (47)
C6A—C1A—C2A—C3A	0.6 (2)	C6A—C1A—C7—O1	156.2 (7)
C7—C1A—C2A—C3A	179.9 (16)	C2A—C1A—C7—O1	−23.0 (15)
C1A—C2A—C3A—C4A	0.3 (2)	C6A—C1A—C7—N1	−21.0 (13)
C15A—O2A—C4A—C5A	0.1 (11)	C2A—C1A—C7—N1	159.8 (7)
C15A—O2A—C4A—C3A	−177.4 (7)	C6A—C1A—C7—C1B	−48 (48)
C2A—C3A—C4A—O2A	176.5 (10)	C2A—C1A—C7—C1B	133 (49)
C2A—C3A—C4A—C5A	−1.0 (5)	N1—N2—C8—C9	178.38 (15)
O2A—C4A—C5A—C6A	−176.6 (11)	N2—C8—C9—C10	178.79 (16)
C3A—C4A—C5A—C6A	0.8 (6)	N2—C8—C9—C14	−2.2 (3)
C2A—C1A—C6A—C5A	−0.9 (5)	C16—O3—C10—C9	−176.08 (16)
C7—C1A—C6A—C5A	179.9 (17)	C16—O3—C10—C11	3.9 (2)
C4A—C5A—C6A—C1A	0.2 (6)	C14—C9—C10—O3	−179.31 (15)
C6B—C1B—C2B—C3B	0.6 (2)	C8—C9—C10—O3	−0.2 (2)
C7—C1B—C2B—C3B	171.5 (18)	C14—C9—C10—C11	0.7 (3)
C1B—C2B—C3B—C4B	0.4 (3)	C8—C9—C10—C11	179.74 (16)
C15B—O2B—C4B—C5B	−4.0 (12)	O3—C10—C11—C12	179.27 (16)
C15B—O2B—C4B—C3B	174.8 (7)	C9—C10—C11—C12	−0.7 (3)
C2B—C3B—C4B—O2B	−179.9 (11)	C17—O4—C12—C11	0.7 (2)
C2B—C3B—C4B—C5B	−1.1 (5)	C17—O4—C12—C13	−179.46 (15)
O2B—C4B—C5B—C6B	179.5 (13)	C10—C11—C12—O4	−179.64 (16)
C3B—C4B—C5B—C6B	0.8 (7)	C10—C11—C12—C13	0.5 (3)
C2B—C1B—C6B—C5B	−0.8 (5)	C18—O5—C13—C14	−0.6 (3)
C7—C1B—C6B—C5B	−171 (2)	C18—O5—C13—C12	179.22 (16)
C4B—C5B—C6B—C1B	0.1 (7)	O4—C12—C13—O5	0.0 (2)
N2—N1—C7—O1	−5.0 (2)	C11—C12—C13—O5	179.89 (16)
N2—N1—C7—C1B	173.2 (12)	O4—C12—C13—C14	179.86 (16)
N2—N1—C7—C1A	172.1 (10)	C11—C12—C13—C14	−0.3 (3)
C6B—C1B—C7—O1	157.6 (8)	O5—C13—C14—C9	−179.94 (16)
C2B—C1B—C7—O1	−12.7 (18)	C12—C13—C14—C9	0.2 (3)
C6B—C1B—C7—N1	−20.6 (15)	C10—C9—C14—C13	−0.4 (3)
C2B—C1B—C7—N1	169.0 (8)	C8—C9—C14—C13	−179.49 (16)
C6B—C1B—C7—C1A	133 (49)		

### Hydrogen-bond geometry (Å, º)

**Table:**

*D*—H···*A*	*D*—H	H···*A*	*D*···*A*	*D*—H···*A*
N1—H1*N*1···O1^i^	0.89 (2)	1.94 (2)	2.8086 (19)	166 (2)
O1*W*—H2*W*1···O5^ii^	0.85	2.36	3.068 (4)	141
O1*W*—H1*W*1···O4^ii^	0.85	2.33	3.036 (5)	141
C6*A*—H6*BA*···O1^i^	0.93	2.55	3.294 (17)	138
C8—H8*A*···O1^i^	0.93	2.49	3.2786 (19)	143

Symmetry codes: (i) *x*, −*y*+1/2, *z*−1/2; (ii) *x*, *y*, *z*−1.
